# Integrated microdevice with a windmill-like hole array for the clog-free, efficient, and self-mixing enrichment of circulating tumor cells

**DOI:** 10.1038/s41378-021-00346-y

**Published:** 2022-02-15

**Authors:** Hao Li, Jinze Li, Zhiqi Zhang, Zhen Guo, Changsong Zhang, Zixu Wang, Qiuquan Guo, Chao Li, Chuanyu Li, Jia Yao, Anran Zheng, Jingyi Xu, Qingxue Gao, Wei Zhang, Lianqun Zhou

**Affiliations:** 1 School of Biomedical Engineering (Suzhou), Division of Life Sciences and Medicine, University of Science and Technology of China, 260026 Hefei, China; 2 CAS Key Lab of Bio-Medical Diagnostics, Suzhou Institute of Biomedical Engineering and Technology, Chinese Academy of Sciences, 215163 Suzhou, China; 3 Ji Hua Laboratory, 528000 Foshan, China; 4 Department of Laboratory Medicine, The Affiliated Suzhou Science and Technology Town Hospital, Nanjing Medical University, 215153 Suzhou, China; 5 Shenzhen Institute for Advanced Study, University of Electronic Science and Technology of China, 518000 Shenzhen, China; 6 Suzhou CASENS Co., Ltd, 215163 Suzhou, China; 7 Jinan Guoke Medical Technology Development Co., Ltd, 250001 Jinan, China

**Keywords:** Biosensors, Microfluidics, Environmental, health and safety issues, NEMS

## Abstract

Circulating tumor cells (CTCs) have tremendous potential to indicate disease progression and monitor therapeutic response using minimally invasive approaches. Considering the limitations of affinity strategies based on their cost, effectiveness, and simplicity, size-based enrichment methods that involve low-cost, label-free, and relatively simple protocols have been further promoted. Nevertheless, the key challenges of these methods are clogging issues and cell aggregation, which reduce the recovery rates and purity. Inspired by the natural phenomenon that the airflow around a windmill is disturbed, in this study, a windmill-like hole array on the SU-8 membrane was designed to perturb the fluid such that cells in a fluid would be able to self-mix and that the pressure acting on cells or the membrane would be dispersed to allow a greater velocity. In addition, based on the advantages of fluid coatings, a lipid coating was used to modify the membrane surface to prevent cell aggregation and clogging of the holes. Under the optimal conditions, recovery rates of 93% and 90% were found for A549 and HeLa cells in a clinical simulation test of our platform with a CTC concentration of 20–100 cells per milliliter of blood. The white blood cell (WBC) depletion rate was 98.7% (*n* = 15), and the CTC detection limit was less than 10 cells per milliliter of blood (*n* = 6). Moreover, compared with conventional membrane filtration, the advantages of the proposed device for the rapid (2 mL/min) and efficient enrichment of CTCs without clogging were shown both experimentally and theoretically. Due to its advantages in the efficient, rapid, uniform, and clog-free enrichment of CTCs, our platform offers great potential for metastatic detection and therapy analyses.

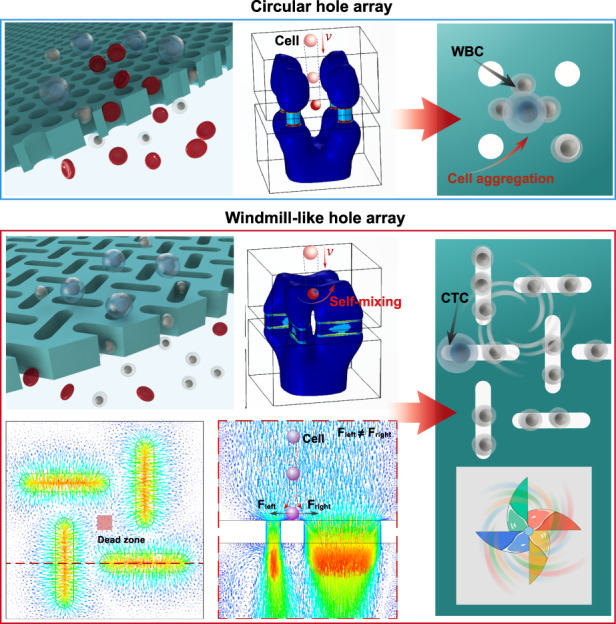

## Introduction

Circulating tumor cells (CTCs), which are shed from a primary tumor and released into the bloodstream, play a seed-like role in the process of cancer metastasis and cause as much as 90% of the cancer-associated mortality^1–3^. The possibility of isolating CTCs from peripheral blood makes it possible for CTCs to serve as a “liquid biopsy” for metastatic tumors and provide cancer-related information in a noninvasive manner^4–6^. Clinical studies have shown that the CTC counts or number of cancer patients is correlated with disease, which can indicate disease progression and evaluate the therapeutic response^7,8^. Based on abundant intrinsic information highly associated with cancer metastasis, the isolation and detection of CTCs is valuable in both clinical research and treatment. Therefore, the development of a new platform is vital and urgent for making the isolation and detection of CTCs more rapid, efficient, and accurate.

Currently, due to the extreme rarity and high heterogeneity of CTCs as well as their vulnerability, the isolation and detection CTCs from whole blood are extremely challenging^9,10^. Various techniques that are mostly based on specific tumor markers (affinity-based strategies) and biophysical properties (label-free strategies) have been developed for the effective isolation and detection of CTCs^9^. Affinity-based capture depends on the immunochemical interactions between specific antigens expressed on the cell surface and their corresponding antibodies immobilized on magnetic beads^11–13^ or patterned structures^14–18^. To date, CellSearch, the only Food and Drug Administration (FDA)-approved platform for CTC detection in breast, prostate and colorectal cancer, has been the gold standard in the field of CTCs^7,19,20^. Nevertheless, due to loss of the epithelial phenotype related to epithelial-mesenchymal transition (EMT) during tumor metastasis^21^, the effect of CTC capture based on epithelial cell adhesion molecular (EpCAM) expression, including CellSearch, has been questioned. In contrast, label-free strategies, with their unique advantages of easy operations and low costs, can capture epithelial and mesenchymal phenotypes, which are more suitable for the critical requirements for clinical application.

There are numerous approaches for CTC capture depending on biophysical properties, such as size and deformability^10,22,23^, electrochemical properties^24,25^, dielectrophoretic separation^26,27^, inertial migration^28,29^, and other characteristics. Nonetheless, among these characteristics, membrane filtration, as a simpler and lower-cost technique based on the difference in size between tumor cells and hematologic cells, has prompted significant interest^30–32^. Such devices consist simply of a three-dimensional configuration and a filter membrane with pores of specific shapes and sizes, which can be flexibly combined with other enrichment strategies^33,34^. Table 1 shows a comparison of the comprehensive performance of membranes based on size filtration over the past 10 years. Several research groups have reported that isolation by size of epithelial tumor cells (ISET) exhibits better performance than CellSearch in clinical tests of patients with various cancers^35–37^. Despite the feasibility of these size-based approaches in clinical applications, their disadvantage, which is that balancing the capture efficiency, purity, and cell viability, is usually difficult, cannot be ignored^38^. For these size-based approaches or devices, the key challenges are clogged pores and cell aggregation, which greatly reduce the selectivity of the CTC isolation process and affect the subsequent fluorescence imaging analysis^10,39^. The polycarbonate filter, which is inexpensive and user-friendly, was first used to enrich CTCs based on size, but randomly distributed or overlapping pores limit the capture efficiency, and the filter tends to become clogged^32,36^. With the development of microfabrication techniques, filters with different types and densities of pores could be precisely manufactured for the enrichment of CTCs^31–34,37,38,40^. Tang et al.^38^ developed a microfluidic device with a microfilter of conical-shaped holes that obtained a higher WBC clearance efficiency (200 or 500 μL/min) than that obtained with cylindrical-shaped holes because the conical shapes prevent the clogging of pores. Furthermore, Hosokawa et al.^41^ reported that the number of WBCs remaining on the microcavity array (MCA) system is significantly reduced by changing the circular array to a rectangular array. Although optimizing the filter microstructure improves the WBC removal efficiency, hole clogging and cell aggregation on the micropores still occurs, which greatly hinders CTC capture and detection. Usually, the sample is diluted to a certain volume to prevent membrane clogging^33,41^. Furthermore, the flow rate, which directly affects the capture efficiency and the uniformity of cell dispersion, is also a key factor in this process^33,38^. As the cells accumulate on the filter membrane, the high flow rate inevitably increases the filtration pressure and applies it to each cell, which greatly increases the possibility that cells are crushed while passing through the filter. In general, a low flow rate ensures the desired effect of these membrane filtration-based approaches or devices, as shown in Table 1. Therefore, the rapid and efficient isolation of CTCs without clogging for membrane filtration still needs further exploration.

**Table 1:** Comparison of the comprehensive performance of membranes based on size filtration over the past 10 years.

Device name	Type of membrane	Size and arrangement of holes	CTC recovery	Purity	Flow rate	Ref.
ScreenCell	Track-etched polycarbonate	7.5 μm circular pores with a random distribution	74% (2 spiked); 91.2% (5 spiked)	\	1.2 mL/min	^32^
Filtration cartridge	Transparent polymer membranes	8 μm circular-pore array	>80%	<1000 WBC contamination	0.1 mL/min	^33^
Microfilter-integrated microfluidic device	Polyethylene glycol diacrylate (PEGDA)	5.5/6.5/8.0 μm conical-hole array	~95%	~96% WBC clearance efficiency	0.2 mL/min	^38^
PMM-integrated microdevice	Polydimethylsiloxane (PDMS) microfiltration membrane (PMM)	9.1 μm circular-pore array	>90%	\	10 mL/h	^48^
Microcavity array (MCA) system	Nickel by electroforming	8 × 30 μm rectangular pores; parallel array	~80%	854 ± 306 recovered WBCs/mL	0.2 mL/min	^41^
Microfluidic platform for negative enrichment	Parylene-C.	5.5 × 40 μm micro slit; parallel array	>90%	2.3 log10 WBC depletion	~0.8 mL/min	^34^
Integrated microdevice with a windmill-like hole array	SU-8	7 × 35 μm windmill-like hole array	93% for A549 cells; 90% for HeLa	98.7% WBC depletion rate	2 mL/min	This work

In this study, a novel integrated microdevice with a windmill-like hole array was developed for the efficient, clog-free and self-mixing enrichment of CTCs. The design of a windmill-like hole array on the SU-8 membrane enabled the cells to be dispersed more evenly and reduced the pressure on the cells. Furthermore, to improve the comprehensive performance of the SU-8 membrane, the surface was modified to avoid clogged pores and cell aggregation, which greatly reduced the number of cell clusters and improved the WBC clearance efficiency. Then, using the high-throughput detection and analysis device, the rapid and accurate identification of CTCs could be achieved. Under the optimal conditions, the advantages of the integrated microdevice for the rapid and efficient enrichment of CTCs without clogging were shown both theoretically and experimentally.

## Design and simulation

**Fig. 1:**
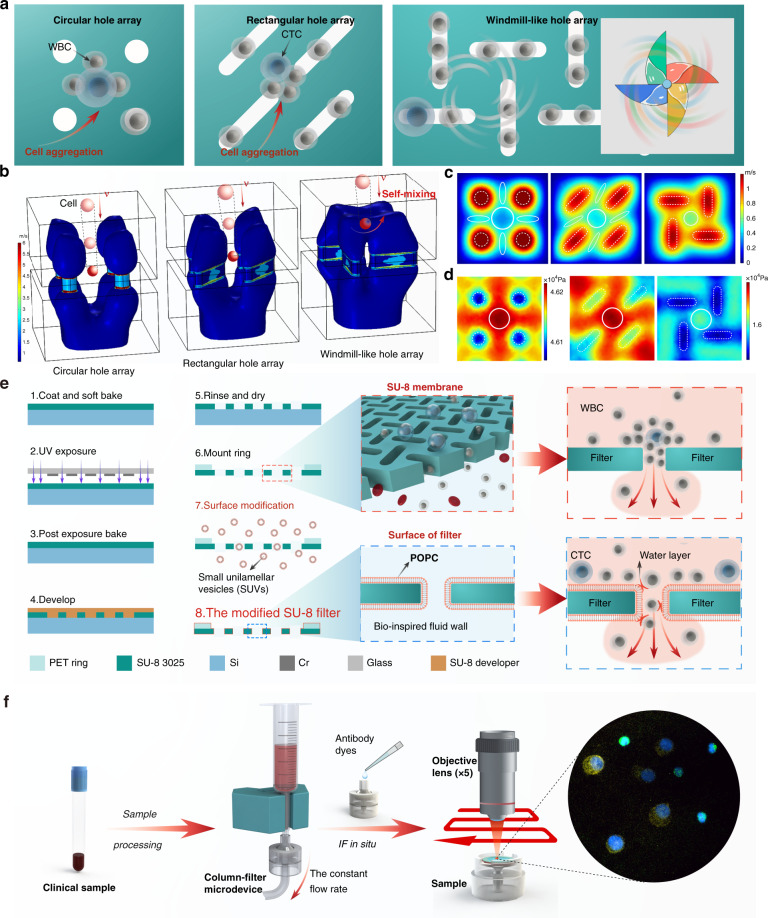
Integrated microdevice with an SU-8 membrane containing a windmill-like hole array for the self-mixing enrichment of circulating tumor cells. **a** Schematic of the cell distribution on membranes with different types of pores. **b–d** Hydrodynamic simulation of steady fluid passing through holes. **b** Three-dimensional contour map of the fluidic velocity field when the fluid passes through three different types of holes (7 μm-diameter circular hole array, 7 × 35 μm rectangular hole array and 7 × 35 μm windmill-like hole array). The fluidic flow within the blue area was relatively rapid (1–6 m/s), whereas the fluidic flow outside the blue area was relatively stable (less than 1 m/s). **c** Horizontal cross-sectional flow field simulation at a height of 15 μm above the holes. **d** Horizontal cross-sectional pressure field simulation at a height of 15 μm above the holes. The white circle is the dead area, where fluid velocity changed little due to the focusing effect of the fluid above the holes. The dotted frame represents the hole array. **e** Fabrication process of the SU-8 membrane with the windmill-like hole array. Using a special microfabrication technique, a homogeneous membrane with a windmill-like hole array could be fabricated accurately. Through surface modification, lipid bilayer-coated microholes on the SU-8 membrane were created to eliminate nonspecific adsorption between cells and the membrane. **f** Schematic of the experimental setup and operation flow. The processed blood sample passed through the separation column and the SU-8 membrane at a constant flow rate, labeled leukocytes were removed in the column, and CTCs were retained on the membrane. After the CTC enrichment process was completed, immunofluorescence was directly performed on the SU-8 membrane.

Hole clogging and cell aggregation still exist, even though filter membranes with microholes of different sizes and shapes and in different arrangements have been microfabricated for CTC enrichment^34,41–43^. To resolve these problems, a windmill-like hole array was designed in this study to perturb the fluid above the microholes, which resulted in homogenization of the distribution of cells in the fluid and a reduced pressure on the cells (Fig. 1a). In nature, a windmill is driven to rotate by the passing airflow, and the airflow is in turn disturbed, which makes the airflow velocity uneven and disperses the pressure generated by the airflow. This phenomenon inspired a novel notion of applying the windmill disturbance model to CTC enrichment. Based on this idea, the microhole structure was first designed as a windmill-like hole array to achieve the clog-free and self-mixing enrichment of CTCs.

Theoretically, when fluid passes through a windmill-like hole array vertically, the fixed windmill-like hole array allows the fluid above the holes to self-mix because the forces are mutual. First, to verify the feasibility of this design, a COMSOL^®^ simulation utilizing simplified boundary conditions and geometric models was performed to compare the fluid dynamics of three types of microhole designs (7-μm-diameter circular hole array, 7 × 35 μm rectangular hole array and 7 × 35 μm windmill-like hole array). A scenario in which fluid flowed vertically at 200 μL/min through the filters (80 × 80 μm) with three different types of holes was simulated. Considering the mechanical strength and utilization of the SU-8 membrane, the center-to-center distance between neighboring holes was initially set to 30 μm. A Reynold’s number of 1.0417 was obtained with the experimental parameters, which showed that the fluid flow in the chamber was generally laminar. To simplify our simulation, we assumed that the fluid was Newtonian and incompressible^44^. The steady fluid flow in the mathematical model obeyed the Navier Stokes equations as follows:

1 $$\rho (u\cdot \nabla )u=\nabla \cdot [-pI+\mu (\nabla u+{(\nabla u)}^{T})]+F$$

2 $$\rho \nabla \cdot u=0\,$$

where *ρ* and *μ* are the density and dynamic viscosity, respectively; *u* and *p* indicate the fluid velocity and fluid pressure, respectively; and *F* is the external force that acts on the fluid.

Because the cells suspended in the fluid (or PBS solution) moved with the flow of the fluid, the status of fluidic flow could roughly reflect the status of cell movement. Figure 1b–d shows hydrodynamic simulations of steady fluid passing through holes. As shown in Fig. 1b, in the three-dimensional contour map of the fluidic velocity field, the fluidic flow within the blue area was relatively rapid (1–6 m/s), whereas that outside the blue area was relatively stable (less than 1 m/s). The area where the fluidic flow was stable was prone to become a dead zone, where cells were easily deposited. The three-dimensional bule area above the windmill-like holes was significantly larger than that above the other two types of holes, which suggested that the cells moved more actively above the windmill-like hole array and had a lower possibility of being deposited on the surface of the dead zone at an equal distance from neighboring holes. Figure 1c shows the horizontal cross-sectional velocity field simulation at a height of 15 μm above the holes. The fluidic velocity in the dead area (the white circle) of the circular holes (0.2–0.4 m/s) and parallel holes (0.4–0.6 m/s) on the horizontal section was significantly lower than that found with the windmill-like holes (~0.8 m/s), which indicated that the circular hole array and rectangular hole array exerted a greater flow-focusing effect on the fluid, and cells were more likely to be deposited on the surface of the dead zone where fluidic flow was not active. Furthermore, to better understand the self-mixing process of the cells above the hole, the trajectory of the fluidic flow was simulated (Supplementary Fig. S1). A micro-vortex was formed above the windmill hole array with a smaller dead zone than the parallel hole array (Supplementary Fig. S1a, b). Due to the horizontal and vertical arrangements of the windmill-like hole array, the distribution of the fluid trajectory in the vertical section was asymmetrical, and the trajectories of cells above the area at an equal distance from neighboring vertical holes were more likely to change compared with that found for neighboring parallel holes before the cells reached the membrane surface. Even if the cell accidentally fell on the middle area of adjacent holes, it would slide into one of the holes because F_left_ ≠ F_right_ (Supplementary Fig. S1c, d). In fact, the self-mixing process of the cells could be reflected by the changes in their trajectory. Therefore, the windmill-like hole array could exert a more obvious disturbing effect on the upper fluid, which would result in the self-mixing of cells.

Moreover, Fig. 1d shows that the partial pressure (the white circle) of the circular and rectangular hole arrays was markedly higher than 1.6 × 10^4^ Pa, whereas the partial pressure of the windmill-like hole array was slightly higher than 1.6 × 10^4^ Pa. For the same inlet flow rate, the pressure above the windmill-like hole array was significantly lower than that above the circular and rectangular hole array. In other words, under the same pressure conditions that the cells could withstand, the flow rate allowed by the windmill-like hole array was significantly higher than that allowed by the other two types of holes. Effectively, this novel design of a windmill-like hole array could homogenize the distribution of cells, prevent holes from clogging, and reduce the pressure on the membrane surface, which would allow a higher flow rate of fluid with cells passing through the holes. In the following sections, SU-8 membranes with different-sized holes based on a windmill-like hole array were fabricated for optimization, and the advantages of this design were further verified through comparisons with different membrane types.

## Results and discussion

### Working principle of an integrated microdevice with a windmill-like hole array

**Fig. 2:**
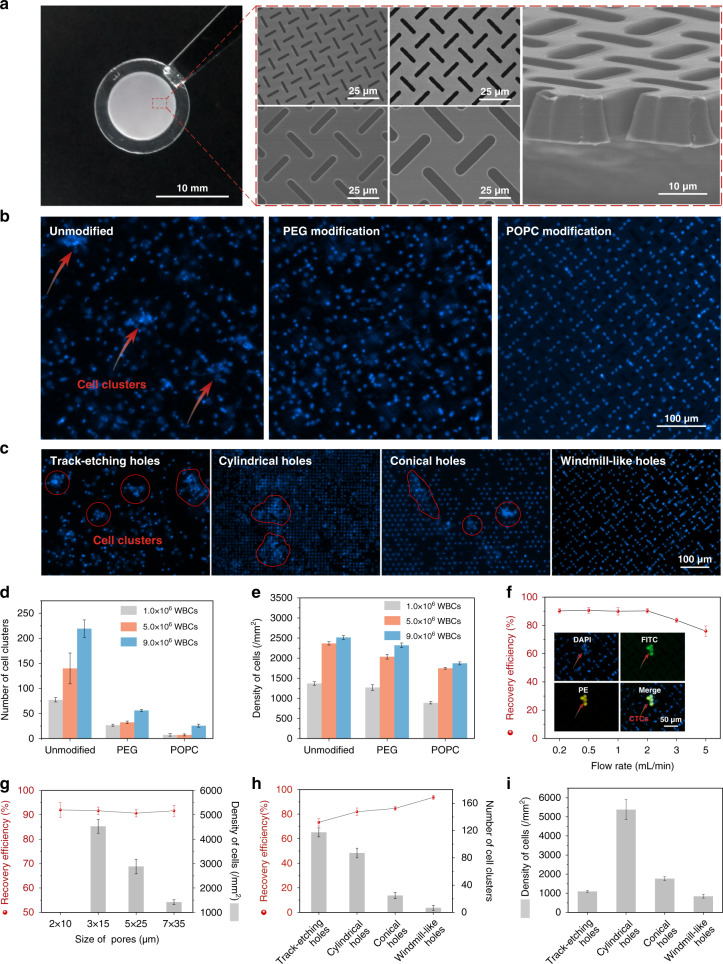
Optimization of the SU-8 membrane and filtering conditions to improve the comprehensive performance of platform. **a** SEM images and physical photograph of the SU-8 membrane. SU-8 membranes with a diameter of 13 mm, an effective diameter of 9 mm and a thickness of 10 μm were obtained with four hole sizes: 2 × 10 μm, 3 × 15 μm, 5 × 25 μm and 7 × 35 μm. **b** Effect of different surface modification conditions on the cell distribution. WBC (1.0 × 10^6^) were spiked in 10 mL of PBS and filtered at 2 mL/min under different conditions (unmodified, PEG and POPC). The red arrows indicate cell clusters. **c** Effect of different types of holes on the cell distribution. WBCs (1.0 × 10^6^) were spiked in 10 mL of PBS and filtered at 2 mL/min using four types of holes (PC with 8 μm-diameter track-etching holes, SU-8 with 7 μm-diameter cylindrical holes, PEGDA with 8-μm conical holes and SU-8 with 7 × 35 μm windmill-like holes). The red circles and arrows indicate cell clusters. **d, e** Effect of different surface modification conditions on the number of cell clusters and the cell density. WBCs (1.0 × 10^6^, 5.0 × 10^6^, and 9.0 × 10^6^) were spiked in 10 mL of PBS and filtered at 2 mL/min under different conditions using SU-8 with 7 × 35 μm windmill-like holes. **f, g** Optimization of conditions for CTC enrichment. The sample contained 4 × 10^6^ WBCs and approximately 400 labeled CTCs. CTCs were accurately identified as DAPI^+^, FITC^+^, and PE^+^. **h, i** Performance comparison among different types of holes under optimal conditions. The sample containing 4 × 10^6^ WBCs and ~400 labeled CTCs was filtered at 2 mL/min. The error bars correspond to the standard deviation from three independent experiments.

A scientific illustration of the developed SU-8 membrane with a windmill-like hole array is depicted in Fig. 1e, and the operation process using the membrane is systematically shown in Fig. 1f. After theoretically verifying the advantages of the windmill-like hole array, homogeneous membranes with pore sizes of 2 × 10, 3 × 15, 5 × 25, and 7 × 35 μm were fabricated accurately using a series of microfabrication techniques (Fig. 2a). Moreover, membranes with the windmill-like hole array could be easily integrated into the syringe filter holder (Supplementary Fig. S2). The processed blood sample passed through the column-filter microdevice at a constant flow rate, labeled leukocytes removed in the column, and CTCs were retained on the membrane. After the CTC enrichment process was completed, immunofluorescence (IF) was directly performed on the membrane. The simple operation process could meet the clinical requirements.

### Surface modification of the SU-8 membrane to reduce nonspecific cell adsorption

Our preliminary experiments showed the formation of cell clusters that caused pore blockages due to the nonspecific adsorption of materials to cells (Fig. 2b). The materials were not specially treated to reduce the nonspecific adsorption. Eventually, the recovery efficiency of CTCs would decrease due to the increased pressure applied to each cell, and it would be difficult for our instrument to identify and analyze all the cells accurately based on cell clusters. To improve the performance of the windmill-like array holes on the SU-8 membrane and meet the requirements of the instrument to accurately identify cells, reducing the nonspecific adsorption between materials and cells was vital. Previous studies have demonstrated that hydrophilicity^45^ including lipid coating^46^ and polyethylene glycol (PEG) modification^47^ could eliminate the nonspecific binding between proteins and material surfaces. Based on previous work, we chose hydrophilic modifications such as 1-palmitoyl-2-oleoyl-sn-glycero-3-phosphocholine (POPC) and PEG to improve the results.

To assess the effect of different modification types on the 7 × 35 μm SU-8 membrane, the number of cell clusters and the cell density under three conditions (unmodified, PEG and POPC) were evaluated. The cell suspension containing different numbers of WBCs was thoroughly mixed to prevent errors caused by adhesion and then added to the device at a flow rate of 2 mL/min. After 4’,6-diamidino-2-phenylindole (DAPI) staining of the filter membrane, the cell distribution was qualitatively observed, and the number of cell clusters and cell density were then quantitatively determined using the high-throughput CTC detection device. Unsurprisingly, remarkable differences in cell uniformity could be observed after the different treatments of the SU-8 membrane (Fig. 2b). Under unmodified conditions, the holes in the filter membrane were prone to blockage and induced the formation of cell clusters that were not conducive to the identification of the instrument. In contrast, the POPC and PEG modifications reduced the occurrence of blockages and made cells uniform according to the hole distribution, which made instrument identification and analysis easy.

Furthermore, we conducted a quantitative analysis of the various modification effects, as shown in Fig. 2d, e. Compared with the effect of the unmodified conditions, both the POPC and PEG modifications reduced the nonspecific adsorption of the filter to a certain extent, but the effect of the POPC modification was significantly better than that of the PEG modification. Specifically, for 1.0 × 10^6^, 5.0 × 10^6^, and 9.0 × 10^6^ WBCs, the number of clusters under the POPC (PEG) modification was reduced by 90.9% (66.2%), 95.0% (77.1%), and 88.1% (74.4%), respectively, compared with that found under unmodified conditions. Moreover, for the same number of WBCs, the lowest cell density was found under the POPC modification. When analyzing the cell density, the instrument excluded the cell clusters formed by multiple cells; therefore, the actual value of the cell density on the unmodified filter membrane was higher than the measured value, but this finding did not affect our comparison of the modification effects. In short, the POPC modification was more conducive to reducing clogging and background interference in the filter membrane.

### Optimization of the conditions for CTC enrichment

After the optimal conditions for WBC depletion that yielded the desired effect were explored (Supplementary Fig. S3), we further optimized the filtration conditions for CTC capture. In the mechanical capture of CTCs, the filtration conditions including the dilution volume, flow rate and filter pore size had certain impacts on the recovery and purity of CTCs^33^. Meunier et al.^33^ proved that the efficiency reached the upper limit with a PBS: blood ratio of 6:1, and dilution was not an effective means to reduce background interference. Therefore, the dilution volume was initially set to 10 mL (>6 mL) to ensure a low transmembrane pressure and a high recovery rate, and the flow rate and filter pore size were optimized to achieve the desired effect. For all optimization experiments, the sample contained 4 × 10^6^ WBCs and ~400 CTCs. In the range of flow rates considered (0.2–5 mL/min with membranes consisting of a 7 × 35 μm hole array), we observed a decreased recovery efficiency when the flow rate was higher than 2 mL/min (Fig. 2f). This finding was obtained because in our system, a flow rate that was too high eluted the cells that magnetically adsorbed on the spheres in the column, which increased the number of cells and the transmembrane pressure on the filter. Finally, filtering at 2 mL/min was identified as the optimal condition for achieving high efficiency and adopted for subsequent experiments. Compared with the other membrane flow rates^33,38^, a higher flow rate (2 mL/min) was needed to achieve the same effect with our system. This finding was obtained because the microstructure on the membrane greatly reduced the transmembrane pressure on the cells, and as a result, the membrane would withstand a greater flow rate without any loss of CTCs (Fig. 1d).

The pore size is expected to be a key factor in the efficiency and purity of CTC enrichment. Hosokawa et al. reported that a rectangular MCA with a size of 8 μm achieved the best balance between high purity and high recovery^41^. Based on this fact, our membrane with a size less than 8 μm was selected for obtaining a higher recovery rate. Evidently, the cell density on the membrane would increase as the size of the rectangular pores decreased. The depletion of upstream WBCs and the high porosity (30–50%) of the SU-8 membrane could reduce the phenomenon that the background interference increased as rectangular pore size decreased. In addition, even if there were many cells on the membrane, all cells, including a few CTCs, were accurately identified by the high-throughput CTC detection device as long as the cells were evenly distributed on the filter membrane without aggregation. As shown in Fig. 2a, we fabricated SU-8 membranes with pore sizes of 2 × 10, 3 × 15, 5 × 25, and 7 × 35 μm for tests. Supplementary Fig. S4 qualitatively shows that the number of cells gradually decreased as the pore size increased. Among the membranes, the membrane with 2 × 10 μm pore sizes had the most cells and the most serious aggregation, which could not be recognized by the instrument (Supplementary Fig. S4a). Figure 2g quantitatively shows the specific recovery rate and cell density obtained with each pore size. With a pore width of less than 8 μm, all recovery rates were higher than 90%; however, the membrane with a pore size of 7 × 35 μm exhibited the lowest background interference, and the cell density was 1423.0 ± 92.7. Therefore, the SU-8 membrane with a pore size of 7 × 35 μm was used as the optimal material for subsequent experiments.

### Performance comparison with the traditional membrane

To further assess the practicability of the SU-8 membrane with a windmill-like hole array, ~400 CTCs spiked in a 1 mL cell suspension containing 10^6^ WBCs were used to evaluate the comprehensive performance of the different types of membranes (PC with 8 μm track-etching holes, SU-8 with 7 μm cylindrical holes, PEGDA with 8 μm conical holes, and SU-8 with 7 × 35 μm windmill-like holes). CTCs were accurately identified by DAPI^+^, FITC^+^, and PE^+^ (Supplementary Fig. S5). By the DAPI staining of nucleated cells, the distribution of cells on different types of membranes was observed, which suggested that the windmill-like hole array on SU-8 could effectively prevent cell aggregation (Fig. 2c).

Furthermore, the recovery rate of CTCs and the number of cell clusters were counted using different types of membranes. As shown in Fig. 2h, compared with other membranes at a flow rate of 2 mL/min, SU-8 with a 7 × 35 μm windmill-like hole array yielded the highest recovery rate (93.7 ± 1.5%) and the lowest number of cell clusters (6.7 ± 3.5). In addition, the cell density on the SU-8 membrane with the windmill-like hole array was the lowest (842.3 ± 96.0), which indicated that the windmill-like hole array could eliminate WBCs with the greatest efficiency (Fig. 2i). In short, the SU-8 membrane with the windmill-like hole array showed advantages for comprehensive performance compared with traditional membranes, which means that subsequent experiments could be conducted based on this finding.

### Validation using simulated clinical samples

**Fig. 3:**
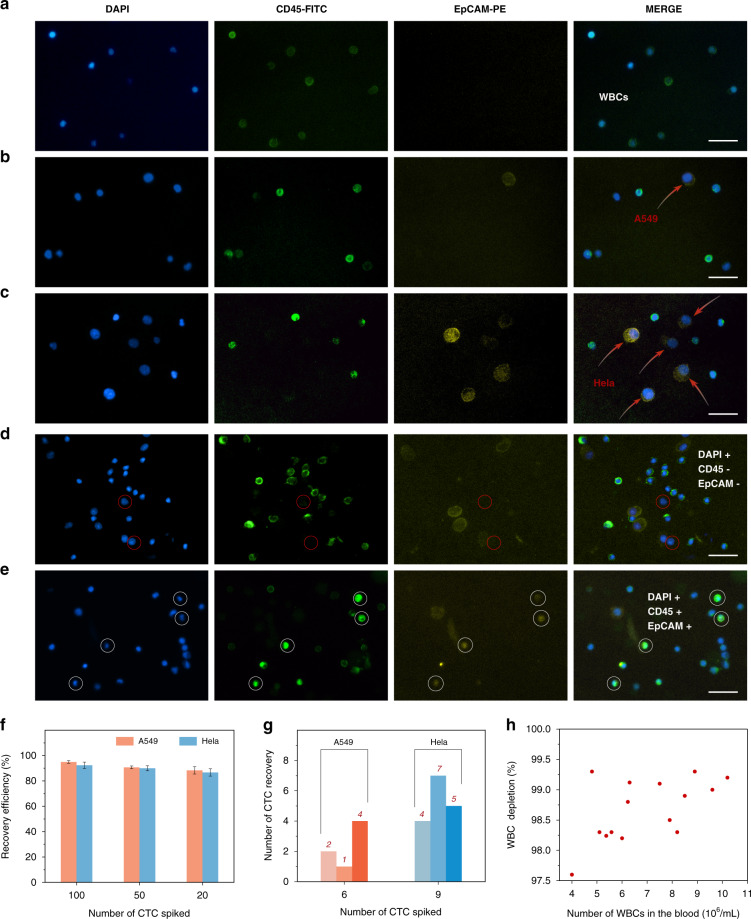
Validation using simulated clinical samples. **a** Fluorescent images of cells captured on the SU-8 membrane after the processing of healthy blood without any spiked cancer cells. **b** Fluorescent images of cells captured on SU-8 membrane after the processing of healthy blood with spiked A549 cells. **c** Fluorescent images of cells captured on SU-8 membrane after the processing of healthy blood spiked with HeLa cells. DAPI^+^, CD45^+^, and EpCAM^−^ cells were considered WBCs. A549 and HeLa cells were identified as DAPI^+^, CD45^−^, and EpCAM^+^. The red arrow indicates CTCs. **d, e** Fluorescent images of positive cells (CD45^+^ and EpCAM^+^) and double-negative cells (CD45^−^ and EpCAM^−^). The scale bars represent 50 μm. **f** CTC recovery efficiencies obtained when 100, 50, and 20 A549 or HeLa cells were spiked into 1 mL of blood. **g** CTC recovery rates obtained when 6.3 ± 2.7 A549 cells or 9.4 ± 2.3 HeLa cells were spiked into 1 mL of blood. The error bars correspond to the standard deviation from three independent experiments. **h** WBC depletion rate from 15 blood samples. Information on the number of WBCs in the blood was obtained from the hospital.

To verify the potential clinical application value of the SU-8 membrane, the recovery efficiency, the WBC depletion rate and the detection limit of the integrated microdevice were comprehensively evaluated under the optimal conditions previously explored. The samples in this set of experiments contained 0–100 live CTCs (A549 and HeLa) and more complex blood from the hospital, which was close to that from cancer patients in the clinic. After sample processing and CTC enrichment, the samples were subjected to IF in situ and identified using a high-throughput detection device (Fig. 1f). Figure 3a–c shows the distinguishing of tumor cells from other cells. Cells that were DAPI^+^, CD45^+^, and EpCAM^−^ were considered WBCs (Fig. 3a). A549 and HeLa cells were identified as DAPI^+^, CD45^−^, and EpCAM^+^ cells (Fig. 3b, c). In addition, during the experiments, a few double-positive cells (CD45^+^ and EpCAM^+^) and double-negative cells (CD45^−^ and EpCAM^−^) were observed (Fig. 3d, e). We speculated that the double-positive cells might be caused by the autofluorescence of the cells due to drying of the sample, and the double-negative cells might be caused by low expression or no expression of EpCAM on CTCs. The cells that we could not judge were all excluded from the CTC enumeration.

It was more challenging to obtain a high recovery efficiency when CTCs were spiked into blood at a low concentration (<100 cells per milliliter of blood) compared with that obtained with a high CTC concentration (1000 cells per milliliter of blood)^41^. When the CTC concentration was low, the loss of one tumor cell would also have a great impact on the recovery efficiency. Therefore, there was a more stringent requirement for the equipment and methods to capture CTCs from samples with a low concentration of CTCs. Regardless of this fact, recovery rates of 95.0 ± 1%, 90.7 ± 1.2%, and 88.3 ± 2.9% were achieved with the spiking of 100, 50, and 20 A549 cells, respectively; and recovery rates of 92.3 ± 2.5%, 90.0 ± 2.0%, and 86.7 ± 2.9% were achieved with the spiking of 100, 50, and 20 HeLa cells, respectively (Fig. 3f). Recovery rates of 93% and 90% were obtained for A549 and HeLa cells, respectively, with a CTC concentration of 20–100 cells per milliliter of blood. Clearly, the fewer the number of cells spiked, the greater the deviation this spiking caused to the results. It was difficult for us to guarantee the actual number when the number of cells spiked was less than 10. The average and standard deviation were calculated to reduce the deviation of the cells spiked from a statistical point of view after counting the number of CTCs contained in 10 μL of the cell suspension five times. As shown in Fig. 3g, we performed three repeated experiments with A549 and HeLa cells. When 6.3 ± 2.7 A549 cells were spiked, we captured 2, 1, and 4 cells; and when 9.4 ± 2.3 HeLa cells were spiked, we captured 4, 7, and 5 cells. In addition, the WBC depletion rate from 15 experiments was recorded, and the average was 98.7% (Fig. 3h). As expected, our platform still obtained a 93% recovery rate for A549 cells and a 90% recovery rate for HeLa cells when the CTC concentration was 20–100 cells per milliliter of blood, the detection limit was less than 10 cells per milliliter of blood, and the WBC depletion rate was 98.7%. By comparing the indicators of our device with the same indicators of other devices in the past decade, our device could not only guarantee a high recovery rate and high purity but also greatly improve the sample processing speed (Table 1). In addition, other advantages, such as reducing clogging and homogenizing the cell distribution, are not quantitatively shown in the table. Considering the desired effect achieved by our device, real clinical samples could be used for a more comprehensive evaluation.

## Clinical test

With ethical approval from the cooperative hospital (Validation date of 2021/08/27, Report No. IRB2021055), blood samples from cancer patients were obtained for clinical trials. A total of 20 blood samples donated by 5 healthy donors and 15 patients with different types of cancer were tested using our integrated microdevice with a windmill-like hole array. The volume of each blood sample was 1–3 mL. The pretreated blood sample was loaded into the column-filter microdevice containing 10 mL of PBS for full dilution before filtration. The filtration experiments were carried out using the SU-8 membrane with a 7 × 35 μm windmill-like hole array at a flow rate of 2 mL/min. In addition, the SU-8 membrane was previously processed by POPC. After filtration and immunofluorescence in situ, fluorescence images of the cells on the membranes were obtained using the CTC high-throughput detection device (blue represents DAPI staining of all the cells, the green color shows CD45^+^, and the yellow color indicates EpCAM^+^).

**Fig. 4:**
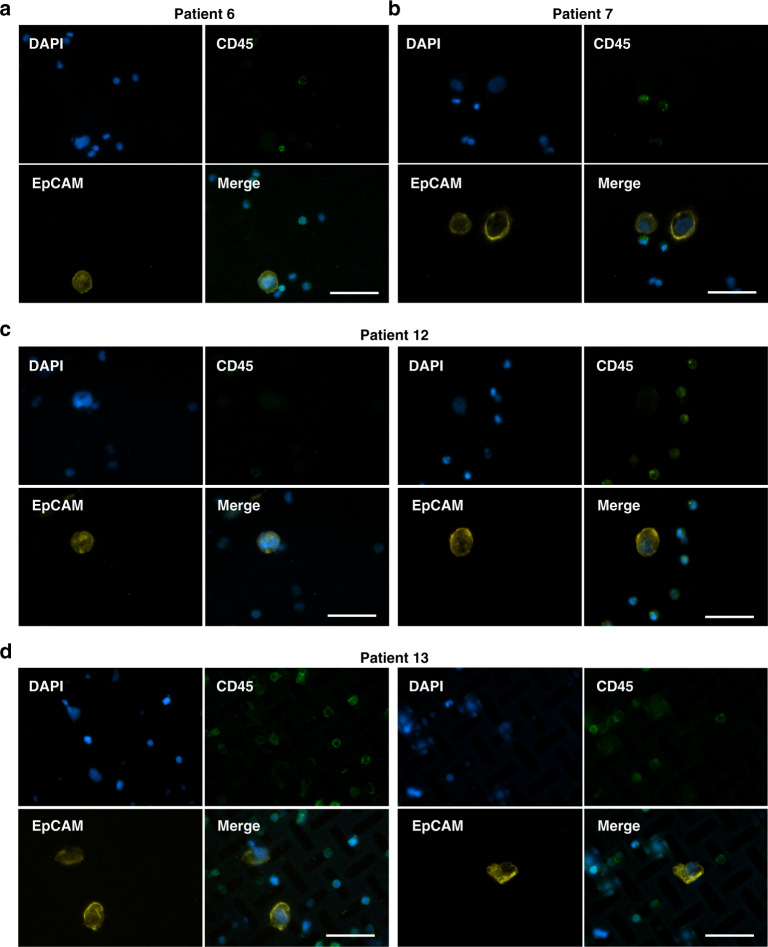
Validation using clinical samples. Four examples of CTC fluorescence images captured from the blood of cancer patient 6 (**a**), patient 7 (**b**), patient 12 (**c**), and patient 13 (**d**). The blue indicates the DAPI staining of all cells, the green color indicates CD45^+^, and the yellow color represents EpCAM^+^. CTCs were identified as DAPI^+^, CD45^−^, and EpCAM^+^. The scale bars represent 50 μm.

As shown in Fig. 4, four examples of CTC fluorescence images captured using the blood from different cancer patients are listed. More fluorescence images of CTCs are shown in Supplementary Fig. S6. EpCAM^+^ and CD45^−^ cells (CTCs) were captured using blood samples from multiple cancer patients, verifying the feasibility of using our platform for CTC isolation and detection. Table 2 shows the test results from all blood samples, including healthy donors and cancer patients. CTCs could hardly be detected in the blood samples from healthy donors, indicating that our device exhibits a low number of false-positives. Statistically, CTCs were identified in 11 of 15 cancer patients (73.3% detection rate); however, no CTC signature was found in the blood of some cancer patients. Two reasons might explain these findings. On the one hand, the epithelial markers such as EpCAM on the CTCs of these cancer patients might be downregulated or lost due to EMT. On the other hand, there might be no CTCs in the sample based on the limited clinical sample volume. In addition, the cells on the membrane were evenly distributed along with the arrangement of the holes, and the particularly obvious phenomenon of cell aggregation and clogged pores was not observed, which greatly improved the accuracy and convenience of detecting captured CTCs using our device (Supplementary Fig. S7). Of course, double-negative cells (CD45^−^ and EpCAM^−^) were still observed on the membrane, but their appearance did not affect our statistical results. Furthermore, further clinical trials should be conducted with more detailed analyses to evaluate whether an integrated microdevice with a windmill-like hole array is a more appropriate tool for the detection of CTCs from metastatic tumors in patients.

**Table 2:** Validation using clinical samples.

No. of patient	Type of cancer	Volume of blood (mL)	Number of CTCs
1	Healthy donor	2	0
2		2	0
3		2	0
4		2	0
5		2	0
6	Liver cancer	1	1
7		1.5	2
8		2.5	4
9		1.5	3
10		2	0
11	Lung cancer	2	0
12		1.2	2
13		1.2	3
14	Breast cancer	3	1
15		2.5	2
16	Stomach cancer	1	4
17		1	2
18	Bladder cancer	1	0
19	Colon cancer	1	0
20	Bile duct tumor	1	1

## Conclusion

In conclusion, an integrated microdevice with an SU-8 membrane containing a windmill-like hole array was developed for the enrichment of CTCs. First, the advantages of the windmill-like hole array design were validated theoretically through comparisons of hydrodynamic simulations with different microhole designs. To improve the comprehensive performance of the windmill-like hole array, different surface modifications were conducted; the POPC modification, which reduced the number of cell clusters by 91.3% and the cell density by 29.0% on average, was considered the best solution. Using a leukocyte suspension spiked with labeled tumor cells, the optimal conditions (flow rate of 2 mL/min and pore size of 7 × 35 μm) for CTC enrichment were applied to obtain the desired effect. Furthermore, it was experimentally demonstrated that the SU-8 membrane with the windmill-like hole array could induce the self-mixing of fluid to prevent pores from becoming clogged and reduce the possibility of cell aggregation compared with that achieved with traditional membranes. Moreover, the clinical practicability of this device was estimated under the optimal conditions using healthy blood samples with 0–100 live tumor cells, and the device achieved a recovery efficiency of 93% for A549 cells, a recovery efficiency of 90% for HeLa cells, a WBC depletion rate of 98.7% and a detection limit of less than 10 cells per milliliter of blood. The advantages of the integrated microdevice for the rapid and efficient enrichment of CTCs without clogging were shown both theoretically and experimentally. Therefore, an integrated microdevice with an SU-8 membrane consisting of a windmill-like hole array has the potential to be a tool for the clinical application and more detailed analyses of CTCs.

## Materials and methods

### Materials and sample preparation

Blood samples from healthy individuals and cancer patients were collected in EDTA-treated tubes at the Affiliated Suzhou Science and Technology Town Hospital, Nanjing Medical University. Isopore^TM^ polycarbonate membranes were purchased from Merck Millipore. PEGDA membranes with 8 μm conical holes were purchased from An Fang Bio (Guangzhou, China). SU-8 membranes with different sizes and syringe filter holders were processed by TopMembranes Technology Company (Shenzhen, China). PBS, fetal bovine serum (FBS), penicillin/streptomycin, RPMI medium 1640, DMEM, 0.25% trypsin-EDTA, 1-step fix/lyse solution (10×), fluorescent molecule labeled or biotin-conjugated antibody, and dimethyl sulfoxide (DMSO) were purchased from Thermo Fisher. Streptavidin-functionalized magnetic beads (2.8 µm) were obtained from BioMag beads (Wuxi, China). A549 and HeLa cells were purchased from Cobioer Biosciences Company (Nanjing, China). Mesophilic-2000 (PEG) was purchased from Meso Biosystems Company (Wuhan, China), and POPC was purchased from Aladdin.

### SU-8 membrane fabrication and surface modification

Figure 1e shows the fabrication and surface modification process of the SU-8 membrane with a windmill-like hole array. First, the silicon substrate was spin coated with a 10-μm-thick negative resist (SU-8 3025) and soft baked at 95 °C for 10 min. A chromium mask was then used to pattern the holes under UV light (180 J/cm^2^), and a postexposure bake occurred directly after exposure. After development, rinsing, drying and mounting of the PET ring, SU-8 organic membranes with a diameter of 13 mm, an effective diameter of 9 mm and a thickness of 10 μm were obtained. Approximately 250,000 microslits (2–7 μm in width and 10–35 μm in length) were arranged vertically and periodically (Fig. 2a). The standard deviation (SD) of the pore diameter was less than 3%, and the porosity was up to 50%. Finally, the SU-8 membrane was soaked in SUVs (POPC)^46^ or PEG solution for surface modification.

### Experimental setup

The experimental setup (column-filter microdevice) was composed of four parts: a separation column, a syringe filter holder, an SU-8 membrane and a peristaltic pump (IPC; Ismatec, Wertheim, Germany). Supplementary Fig. S2 shows the specific structure of the syringe filter holder. The processed blood sample passed through the separation column and the SU-8 membrane at a constant flow rate provided by the peristaltic pump, labeled leukocytes were removed from the column, and CTCs were retained on the membrane. After the negative enrichment process was completed, the subsequent procedures, including washing, blocking, and immunofluorescence, were directly performed for the SU-8 membrane. Figure 1f shows a schematic representation of the experimental setup and operating flow.

### Cell culture and cell spiking

A549 cells were cultured in RPMI 1640 containing 10% FBS and 1% penicillin/streptomycin. HeLa cells were cultured in basic DMEM supplemented with 10% FBS and 1% penicillin/streptomycin. All cell lines were grown and maintained at 37 °C with 5% CO_2_ in a humidifiedatmosphere. The cells were harvested with 0.25% trypsin-EDTA and resuspended in PBS before use.

In the preliminary optimization experiment, only 400 labeled CTCs were mixed with a WBC suspension from blood to optimize the parameters. To simulate the actual situation of clinical cancer patients and verify the practicability of the platform, 10 μL of a suspension of A549 or HeLa cells (10, 20, 50, or 100 CTCs per 10 μL) was spiked into 1 mL of blood for testing under the optimal conditions. In addition, the exact number of cells spiked into the blood was controlled by counting them multiple times on a somatic cell counting slide (Citotest; Suzhou, China).

### CTC isolation

Clinical samples or simulated samples (blood mixed with CTCs) needed to be preprocessed before CTC isolation. One milliliter of the blood sample was lysed and fixed for 15 min with 10 mL of one-step fix/lyse solution (1×) and then centrifuged to retain the cell pellet. The sample was diluted with 200 μL of PBS, specifically labeled with 2.5 μL of anti-human CD45 biotin and incubated for 20 min. The sample was then incubated for 15 min with 2.5–20 μL of streptavidin- magnetic beads (10 mg/mL). Before sample introduction, the column-filter device was rinsed and filled with PBS. The processed sample was then added to the device and mixed well with PBS. The diluted sample was driven to pass through the column-filter device at a stable flow rate by the negative pressure generated by the peristaltic pump. In addition, the flow rate could be controlled by changing the peristaltic pump’s speed. When the sample was reduced to the filling height of the steel ball, PBS was introduced to rinse the device.

### Cell immunostaining

After microfiltration of the sample solution, the separation column was removed, and the captured cells on the SU-8 membrane were treated inside the syringe filter holder. Two hundred microliters of 0.2% Triton X-100 in PBS was introduced to the filter holder and incubated for 5 min to permeabilize cells, and 200 μL of PBS was then introduced at 50 μL/min to rinse the sample. Similarly, the sample on the membrane was incubated for 5 min with 200 μL of 1% BSA in PBS to block nonspecific binding and rinsed with 200 μL of PBS at 50 μL/min. The cells on the membrane were then incubated with 200 μL of immunostaining solution containing anti-EpCAM PE (1:40 dilution) and anti-human CD45 FITC (1:80 dilution) at 4 °C in the dark for 30 min and rinsed with 200 μL of PBS at 50 μL/min. After staining with 1 μg/mL DAPI for 8 min, the sample was rinsed with 200 μL of PBS at 50 μL/min. The screw cap, gland, and silicone gasket were then carefully removed from the syringe filter holder, and 10–20 μL of ProLongTM Diamond Antifade Mountant with DAPI (Thermo Fisher) was dropped on the membrane. Finally, a cover glass was carefully mounted to avoid air bubbles.

### Cell identification and statistical analysis

The sample was placed into the injection port of the high-throughput detection and analysis device that we previously built. The automatic stage moved in the direction set by the program to scan through the entire membrane, which yielded DAPI, FITC, and PE fluorescence images. The Spinnaker SDK software that we wrote was used to stitch all the acquired cell images and complete cell counting and analysis. To ensure the accuracy of CTC counting, experienced cell biologists reanalyzed all fluorescence images artificially, and counted the number of CTCs after analyzing them with the software. In addition, the CTC recovery, cell density, and WBC depletion were obtained as follows:

3 $${\mathrm{Recovery}}\,{\mathrm{efficiency}}\,( \% )=\frac{{N}_{R}}{{N}_{S}}$$

4 $${\mathrm{Density}}\,{\mathrm{of}}\,{\mathrm{cells}}=\frac{{C}_{R}}{{S}_{E}}$$

5 $${\mathrm{WBC}}\,{\mathrm{depletion}}\,( \% )=\frac{{C}_{S}-{C}_{R}}{{C}_{S}}$$

where *N*_*R*_ and *N*_*S*_ are the number of CTCs captured on the membrane and the total number of CTCs, respectively, spiked into the leukocyte suspension or blood, respectively; *C*_*R*_ and *C*_*S*_ are the number of cells retained on the membrane and the total number of cells spiked, respectively; *S*_*E*_ is the effective area of the filter membrane; and *N*_*R*_ and *C*_*R*_ were directly counted by a high-throughput detection and analysis device.

## Supplementary information

Integrated microdevice with the windmill-like hole array for clog-free, efficient and self-mixing enrichment of circulating tumor cells - Supplementary materials
