# Supplementary material for: Integrated microdevice with a windmill-like hole array for the clog-free, efficient, and self-mixing enrichment of circulating tumor cells
Source: Microsyst Nanoeng. 2022 Feb 15;8:23. doi: 10.1038/s41378-021-00346-y (PMC8844004; doi:10.1038/s41378-021-00346-y)
Supplement: Supplementary file 1 — Integrated microdevice with the windmill-like hole array for clog-free, efficient and self-mixing enrichment of circulating tumor cells - Supplementary materials [file 41378_2021_346_MOESM1_ESM.pdf]

Integrated microdevice with the windmill-like hole array for clog-free, efficient and self-mixing enrichment of circulating tumor cells

Supplementary materials

Design and simulation

To better understand the self-mixing process of the cells above the hole, the trajectory of the fluidic flow was simulated (**Supplementary Fig. S1**). It has been previously reported that the effect of the rectangular hole array was better than that of the circular hole array[1]. Here we did not perform simulation verification on the circular hole array. The micro-vortex could be observed above the windmill hole array with a smaller dead zone than the parallel hole array, which made the cells above the hole in a self-mixing state and not easy to deposit on the area of adjacent holes (**Supplementary Fig. S1a, b**). Due to the horizontal and vertical arrangement of the windmill-like hole array, the distribution of the fluid trajectory in the vertical section was asymmetrical (**Supplementary Fig. S1c, d**). Before the cells reached the membrane surface, the trajectories of cells above area at equidistance of neighboring vertical holes were more likely to change compared to that of neighboring parallel holes. Even if the cell accidentally fell on the middle area of adjacent holes, it would slide into one of the holes on account of $F_{left} \neq F_{right}$. In fact, the self-mixing process of the cell could be reflected by the change of its trajectory. Therefore, the windmill-like hole array could have a more obvious disturbing effect on the upper fluid thereby making the cell self-mix.

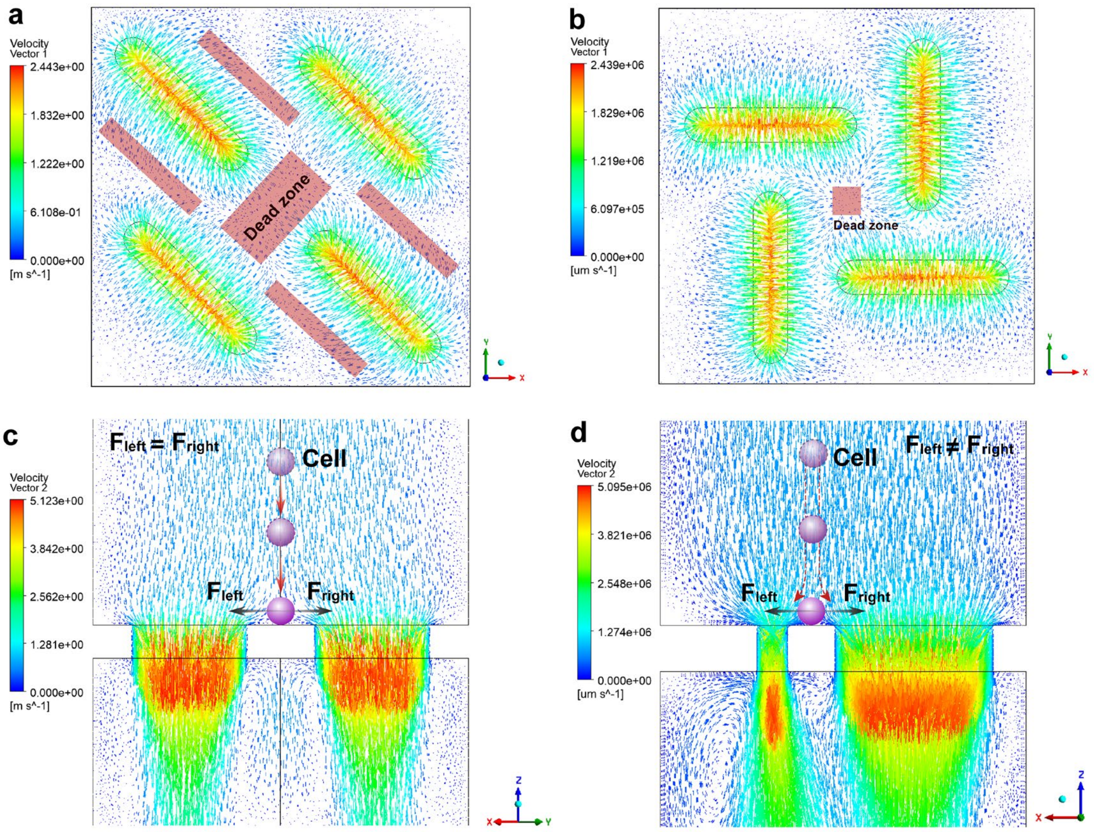

Fig. S1 The trajectory of the fluidic flow above the parallel hole array and the windmill-like hole array. a, b) The trajectory of the fluidic flow in the horizontal section of the parallel hole array and the windmill-like hole array. **c, d)** The trajectory of the fluidic flow in the vertical section of the parallel hole array and the windmill-like hole array. The red areas represent the dead zones.

Syringe filter holder design

Syringe filter holder we designed could be used as a transition device connecting upstream leukocyte depletion and downstream CTC capture. Polypropylene (PP) was used for structural material due to its translucency and chemical resistance. Syringe filter holder was composed of gland, support sheet, silicone gasket, screw cap and base. The specific structure was shown in the Fig. S2. The gland inlet was designed as a standard interface to facilitate external insertion of the separation column to achieve the purpose of continuous sample processing without manual sample transfer. The precision-manufactured rectangular slit membrane was placed on a 13 mm filter support sheet. The combination of the gland and the silicone gasket converted the rotating force

of the screw cap into pressure, which could avoid the deformation of the filter membrane under the premise of sealing. Moreover, gland and silicone gasket ensured a space of 300 μL above the filter membrane, which can save the amount of antibody reagents in the subsequent staining steps.

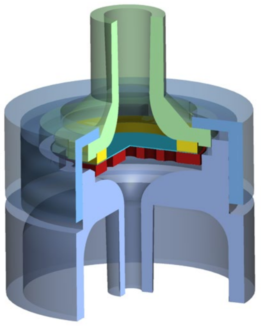

Fig. S2 Schematic of the syringe filter holder's specific structure

Optimization of conditions for CTC enrichment

Negative enrichment which is the preferred method for CTC isolation does not depend on the expression of biomarkers, thereby avoiding the loss of CTC caused by low or non-expression of biomarkers[2]. The feasibility of CD45-functionalized magnetic beads was first verified to remove WBCs. CD45 antibodies and magnetic beads were coupled by the combination of streptavidin and biotin, which was simple and rapid (Fig. S3a). Different from the combination of biotinylated CD45 and streptavidin magnetic beads, the binding of CD45 antibody and WBCs depended not only on the CD45 expression of leukocytes, but also on the harsh incubation system. Considering that the magnetic bead solution complicated the antibody incubation solution in our operating system, we first labeled WBCs with biotinylated CD45 and then coupled the CD45 with streptavidin magnetic beads to ensure a purified incubation system instead of first labeling streptavidin magnetic beads with biotinylated CD45. Fig. S3a shows the details of the negative enrichment sample processing. In addition, 10 μL from the magnetic bead incubation solution was dropped on the glass slide, and observed it under the microscope in bright field. Fig. S3b shows a representative brightfield micrograph, which includes WBCs, CTCs, and WBC labeled with magnetic beads distinguished by size. It confirms that CD45 magnetic beads could specifically label WBCs to ensure

that subsequent experiments could be carried out through our method.

The optimal conditions for WBC depletion were explored to get desired leukocyte removal rate without reducing the CTC recovery rate, including amount of magnetic beads and magnetic beads incubation volume. In all experiments, the amount of biotinylated CD45 antibody for labeling WBCs was 2.5 μg, much higher than the standard amount to ensure that white blood cells were labeled as many as possible. When the amount of magnetic beads was 0.15 mg, the WBC depletion rate (defined as the number of WBCs removed by magnetic separation divided by the total number of WBCs) reached the upper limit, which was 57.6±2.8% (Fig. S3c). The incubation volume of magnetic beads affected the concentration of magnetic beads directly, and higher concentrations of magnetic beads reduced the recovery rate of CTC. It was found that when the incubation volume was more than 4 mL (magnetic bead concentration was less than 0.0375mg/mL), the CTC recovery rate was more than 85% and tended to be stable (Fig. S3d). Therefore, under the premise of ensuring the recovery rate of CTC, 0.15 mg of magnetic beads and 4mL of incubation volume were the optimal conditions to remove WBCs at maximum efficiency.

Pore size is expected to be a key factor in the efficiency and purity of CTC enrichment. Fig. S3 shows the distribution of cells on SU-8 membranes with pore sizes of 2×10 μm, 3×15 μm, 5×25 μm, and 7×35 μm. Not surprisingly, the number of cells gradually decreased as the pore size increased. Among them, the membrane of 2×10 μm had the most cells and the aggregation was the most serious, which could not be recognized by the instrument (Fig. S3a).

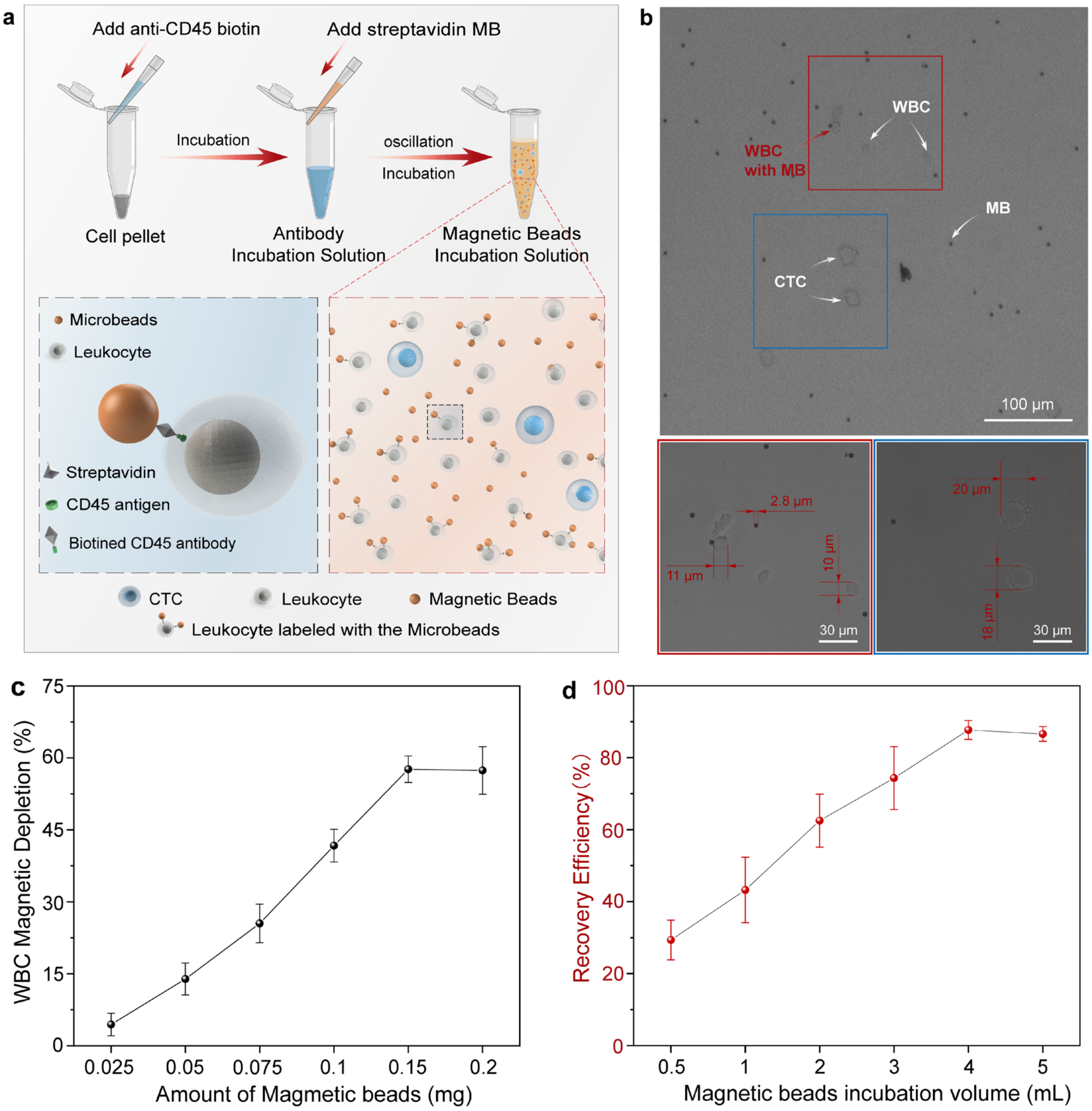

Fig. S3 Negative enrichment strategy for CTC enrichment. a Schematic of sample preparation procedures. **b** Representative brightfield micrograph including WBCs, CTCs, and WBC labeled with magnetic after being incubated with CD45-functionalized magnetic beads. **c** Effect of amount of magnetic beads on WBC magnetic depletion. **d** Effect of magnetic beads incubation volume on recovery efficiency

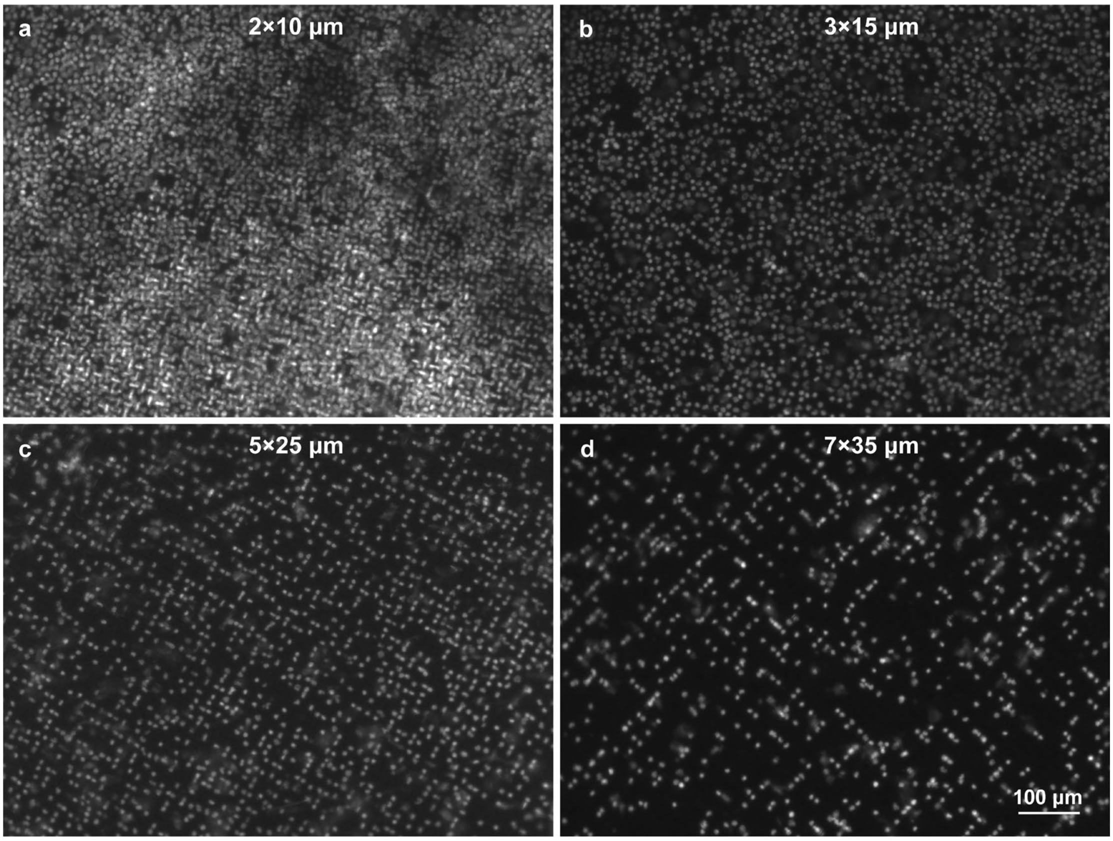

Fig. S4 Qualitative comparison of cell distribution with different sizes of holes on the same experimental conditions

Performance comparison with traditional membranes

To evaluated the comprehensive performance of different types of membrane, approximately 400 labelled CTCs were spiked in 10^6 WBCs suspension. As shown in Figure S5, CTCs on different types of membrane are accurately identified by $DAPI^+$, $FITC^+$ and PE^+. Clearly, the most uneven distribution of cells on the PC membrane can be observed, which is likely to occur cell aggregation. In addition, we quantitatively counted the cell density on the four types of membrane, and result shows that the cells retained on the SU8 membrane are the least (842.3±96), as shown in Fig. S5.

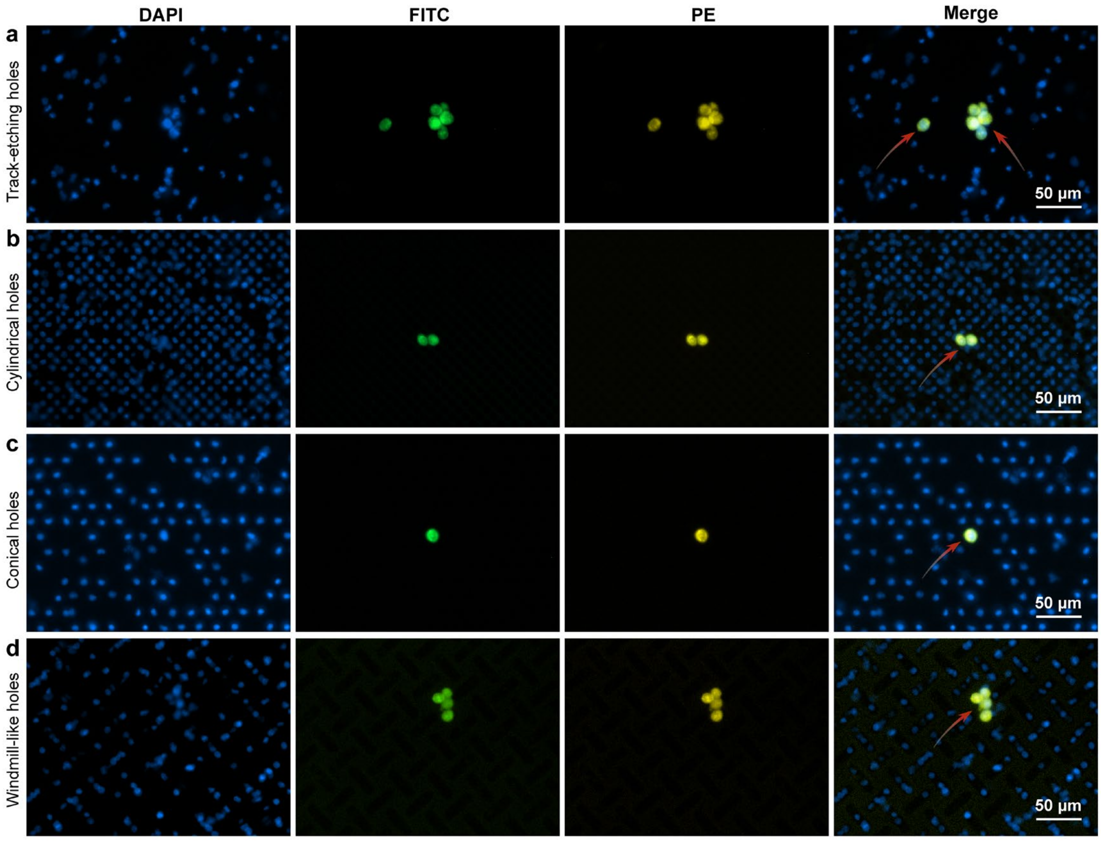

Fig. S5 Fluorescent images of cells captured on the different types of membrane. The red arrow indicates CTC.

Clinical test

For the clinical evaluation, blood samples donated by 5 healthy donors and 15 patients with different type of cancer were used in the study. The volume of each blood sample was 1~3 mL. The pretreated blood sample was loaded into the column-filter microdevice containing 10 mL of PBS for full dilution before filtration. The filtration experiments were carried out on the SU-8 membrane of 7×35 μm windmill-like hole array at a flow rate of 2 mL/min. In addition, the SU-8 membrane was processed by POPC in advance. After filtration and immunofluorescence in situ, fluorescence images of the cells on the membranes were obtained by the CTC high-throughput detection device (blue for DAPI of all cells, green for CD45+ and yellow for EpCAM+).

Fig. S6 shows CTC fluorescence merged images captured with the blood from other seven cancer patients, indicating the feasibility of CTC isolation and detection on our platform. Clearly, the cells on the membrane were evenly distributed along with the arrangement of the holes and the particularly obvious phenomenon of cell aggregation

and clogged pores did not show up (Fig. S7). These results of clinical trials indicated the feasibility of CTC isolation and detection on our platform. Moreover, further clinical trials should be conducted with more detailed analyses to evaluate whether our device is a more appropriate tool for CTC detection of metastatic tumors in patients.

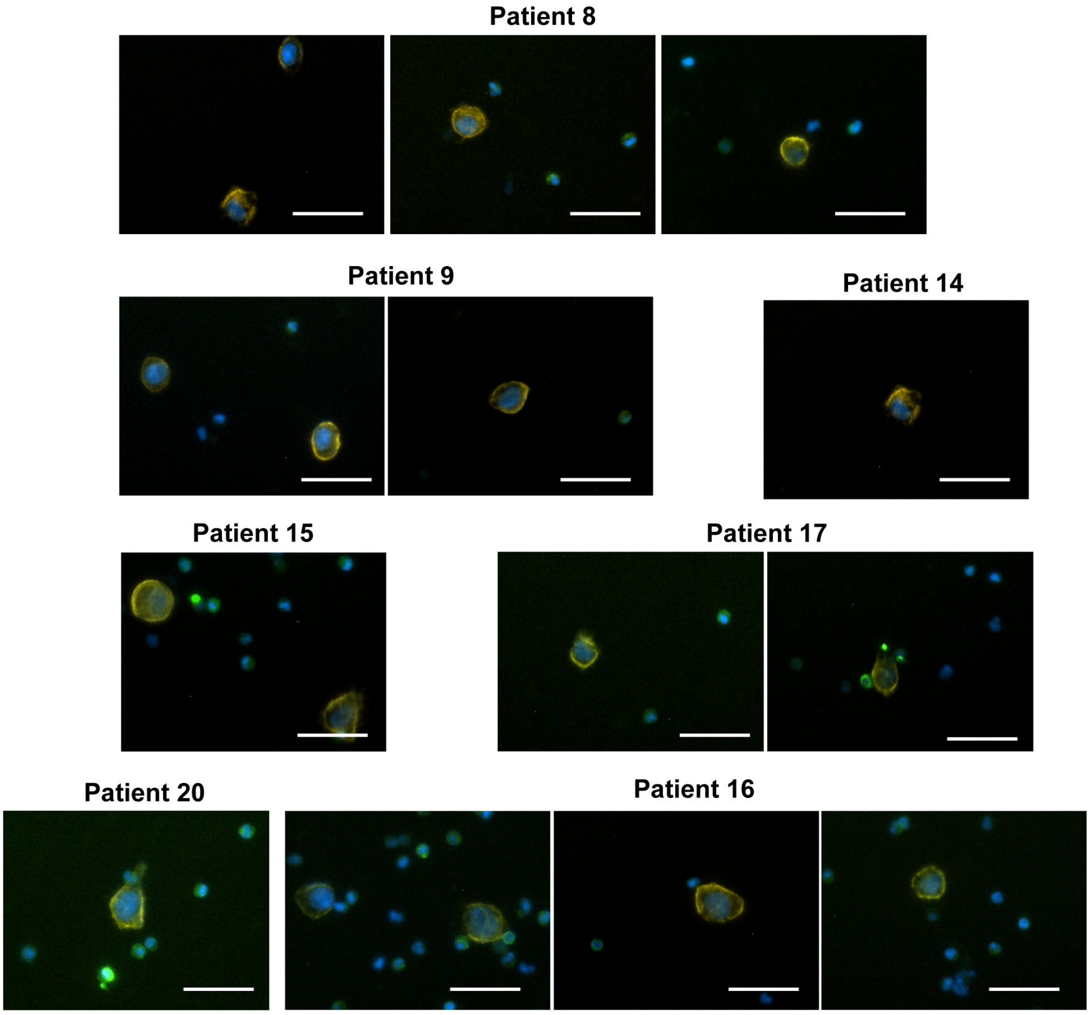

Fig. S6 CTC fluorescence merged images captured with the blood from other seven cancer patients. Scale bars represented 50 μm.

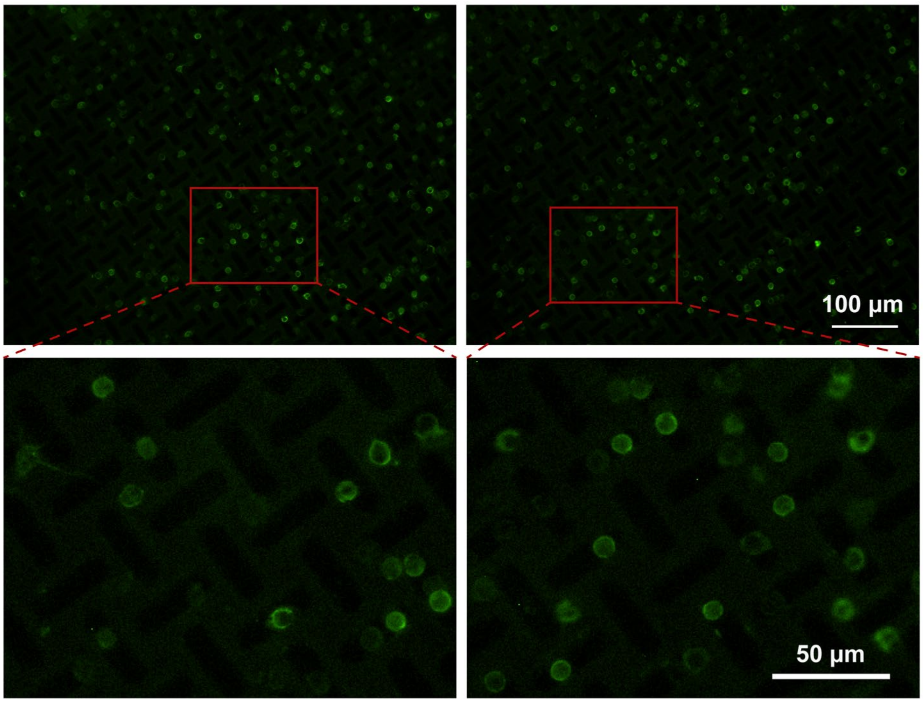

Fig. S7 Cell distribution from clinic sample.

Reference

1 Hosokawa, M. *et al.* Microcavity array system for size-based enrichment of circulating tumor cells from the blood of patients with small-cell lung cancer. *Anal. Chem.* **85**, 5692-5698 (2013).

2 Sajay, B. N. *et al.* Microfluidic platform for negative enrichment of circulating tumor cells. *Biomed. Microdevices* **16**, 537-548 (2014).
